# Transport of Designed Ankyrin Repeat Proteins through reconstituted human bronchial epithelia and protection against SARS-CoV-2

**DOI:** 10.1038/s41598-023-32269-1

**Published:** 2023-04-04

**Authors:** Lisa Künzi, Sarah Ryter, Andreas Cornelius, Zaira Leni, Nathalie Baumlin, Matthias Salathe, Marcel Walser, Olivier Engler, Marianne Geiser

**Affiliations:** 1 Institute of Anatomy, University of Bern, 3012 Bern, Switzerland; 2 Labor Spiez, Federal Office for Civil Protection, 3700 Spiez, Switzerland; 3 Molecular Partners AG, 8952 Zürich-Schlieren, Switzerland; 4 Department of Internal Medicine, University of Kansas Medical Center, Kansas City, Kansas 66160 USA

**Keywords:** Drug development, Viral infection, Protein translocation

## Abstract

Clinical studies have proven antiviral effectiveness of treatment with a Designed Ankyrin Repeat Protein (DARPin) specific against the spike protein of severe acute respiratory syndrome coronavirus type 2 (SARS-CoV-2). More information on transport mechanisms and efficiency to the site of action is desirable. Transepithelial migration through air–liquid interface (ALI) cultures of reconstituted human bronchial epithelia (HBE) was assessed by Enzyme-Linked Immunosorbent Assays and Confocal Laser Scanning Microscopy for different DARPin designs in comparison to a monoclonal antibody. Antiviral efficacy against authentic SARS-CoV-2, applied apically on HBE, was investigated based on viral titers and genome equivalents, after administration of therapeutic candidates on the basal side. Transepithelial translocation of all DARPin candidates and the monoclonal antibody was efficient and dose dependent. Small DARPins and the antibody migrated more efficiently than larger molecules, indicating different transport mechanisms involved. Microscopic analyses support this, demonstrating passive paracellular transport of smaller DARPins and transcellular migration of the larger molecules. All therapeutic candidates applied to the basal side of HBE conferred effective protection against SARS-CoV-2 infection. In summary, we have shown that DARPins specific against SARS-CoV-2 translocate across intact airway epithelia and confer effective protection against infection and viral replication.

## Introduction

Since the beginning of the coronavirus disease 2019 (COVID-19) pandemic, there have been hundred millions of confirmed cases and millions of deaths reported world wide^1^. Vaccination campaigns and immunization against severe acute respiratory syndrome coronavirus type 2 (SARS-CoV-2) have significantly altered the dynamics of the COVID-19 pandemic to transition towards an endemic disease. However, there are currently still about 0.3 to 0.4 million new COVID-19 cases reported world-wide every 24 h^1^, and the last months have shown that the situation is still not stable. A considerable part of the world’s population remains non-immunized^2^ and a previous immunization does not prevent from contracting SARS-CoV-2^3^. New SARS-CoV-2 variants will continue to evolve with unknown potential for new pandemic outbreaks. Moreover, health care systems around the world are under considerable pressure. Thus, there is pressing need for globally accessible therapeutics to treat patients with severe COVID-19, and to protect health care workers and individuals with underlying medical conditions that preclude them from being vaccinated or developing a strong and lasting immune response against SARS-CoV-2.

Amongst other antiviral treatments, ensovibep, a Designed Ankyrin Repeat Protein (DARPin) therapeutic candidate was recently reported as a potential new treatment option with activity against many emerging SARS-CoV-2 variants of concern^4^. Positive efficacy data were reported in early-stage ambulatory patients in the EMPATHY Phase II trial^5^ using ensovibep. In contrast, the treatment with ensovibep in a clinical trial with late-stage hospitalized patients did not result in a significant patient benefit, comparable to the other antiviral biologics tested with the same study set-up^6–9^.

As a distinct class of binding proteins, DARPins are designed from natural ankyrin repeats and are eliminated via kidney clearance within a few minutes^10^. Human Serum Albumin (HSA)-binding DARPin domains were described to prolong the systemic half-life of DARPins to several weeks of circulation in the human body^11^. The underlying principle is serum albumin recycling via the neonatal Fc receptor (FcRn), which is also used by antibodies to prolong their systemic circulation^12^. There are promising pre-clinical and clinical data for the DARPin candidate ensovibep, however little is known regarding both the mechanism(s) and the efficacy by which DARPin molecules pass the pulmonary epithelium. Antibodies are known to be transported across epithelial barriers by receptor mediated transcytosis, i.e. after binding to specific membrane receptors (FcRn for immunoglobulin G (IgG) and polymeric immunoglobulin receptor (pIgR) for both IgM and IgA), they are taken up by the cells via clathrin-mediated endocytosis^13,14^. As mentioned above, albumin also binds to FcRn, raising the question whether albumin binding via HSA-binding DARPin domains enhances their ability to pass through intact airway epithelia.

In this study, we investigated the migration of different DARPin molecules through air–liquid interface (ALI) cultures of reconstituted human bronchial epithelia (HBE) in comparison to the anti-SARS-CoV-2 monoclonal antibody (mAb) Casirivimab (REGN10933). We investigated DARPin molecules varying in designs and sizes, and applied them to the basal side of HBE in the presence or absence of bound HSA. We also assessed the antiviral efficacy of a multispecific DARPin candidate, ALE058, which targets different domains of SARS-CoV-2^15^, Casirivimab and Remdesivir.

## Results and discussion

### Quantitative transepithelial transport of DARPin molecules

**Figure 1:**
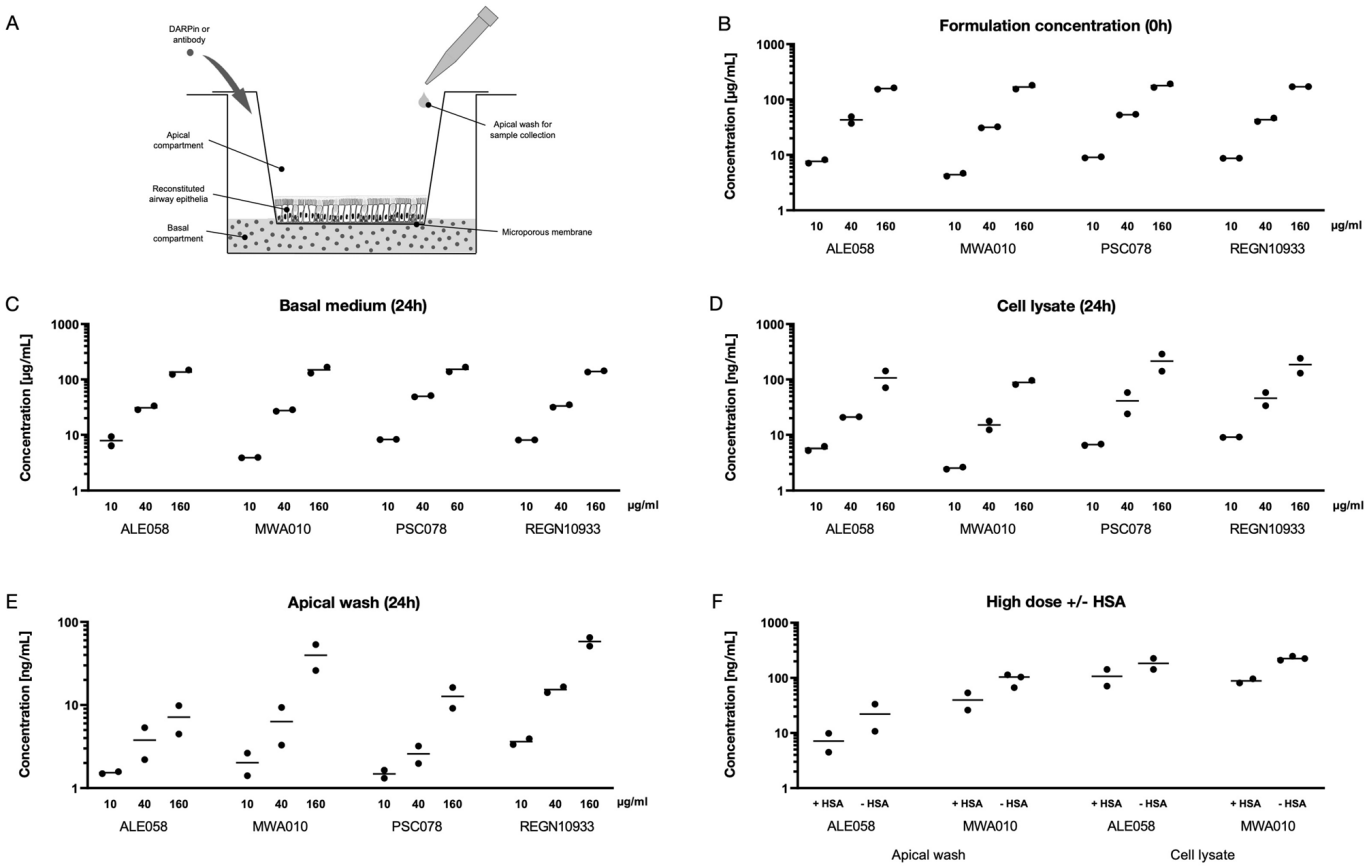
Quantification of DARPin molecules and control antibody in cell lysates, apical washes and basal cell culture medium by ELISA. (**A**) Illustration of the test system used to quantify transepithelial migration of DARPins and control antibody. HBE were reconstituted from cells isolated from a normal donor lung grown on microporous filter inserts at the air–liquid interface. Fully differentiated HBE comprise of ciliated, secretory and basal cells, and produce their own liquid lining layer. The test compounds were added to the basal cell culture medium. Transepithelial migration was assessed by collecting apical washes and lysis of the HBE cell layer after 24 h. (**B**) Verification of the formulation concentrations (10, 40 and 160 µg/mL) added to the basal cell culture medium at 0 h. (**C–E**) Concentration of DARPins and control antibody at 24 h in (**C**) the basal cell medium, (**D**) the HBE lysates and (**E**) the apical washes. (**F**) Concentrations determined for the DARPin candidates ALE058 and MWA010 in apical washes and in HBE lysates at 24 h. Both HSA-binding DARPin candidates were applied to the basal compartment at a concentration of 160 µg/mL in presence or absence of HSA.

The five domain DARPin ALE058 with the ability to neutralize the SARS-CoV-2 spike protein, two control DARPins, which do not bind to SARS-CoV-2 (MWA010 and PSC078, composed of different numbers of binding domains and, hence, of different sizes) and the anti-SARS-CoV-2 mAb REGN10933 were added to the basal medium of HBE cultures (Fig. 1A) to study their transepithelial migration. Detailed information on the different DARPin constructs can be found in Table 1 and the methods section. The Enzyme-Linked Immunosorbent Assay (ELISA) measurements confirmed the concentrations of all compounds in the basal cell culture medium to be in the range of the envisioned 10 µg/mL (low), 40 µg/mL (medium) and 160 µg/mL (high) at the time of inoculation (0 h, Fig. 1B), and to remain comparable to the added formulation concentrations 24 h later (Fig. 1C). The measurements in cell lysates (Fig. 1D) and in apical washes (Fig. 1E) at 24 h post inoculation confirmed that all four investigated compounds passed the intact bronchial epithelium in a concentration dependent manner. For all four compounds, higher concentrations added to the basal medium resulted in higher concentrations in both the cell lysates and the apical lining layer washes. While the concentrations of the test compounds were comparable in the cell lysates (Fig. 1D), they differed in the apical washes (Fig. 1E). The highest values were obtained for the monoclonal antibody (REGN10933), with a median of 58 ng/mL at the highest concentration. The corresponding concentrations of the DARPin candidates were 40 ng/mL for MWA010, 13 ng/mL for PSC078 and 7 ng/mL for ALE058. We additionally evaluated the impact of HSA complexed to the HSA-binding DARPin domains of the candidates MWA010 and ALE058 at their highest concentration of 160 µg/mL. For both DARPin candidates, the concentrations in the cell lysates and in the apical washes were 2–4 times higher, when there was no HSA added to the cell culture medium (Fig. 1F).

**Table 1:**
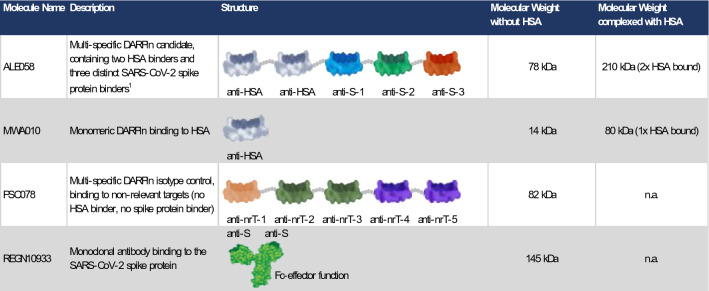
Description of the DARPin candidates and the monoclonal antibody.

^1^Multi-Mode DARPin candidate described in Walser et al. ^11^.

*HSA* human serum albumin, *S* SARS-CoV-2 spike protein, *nrT* non-relevant target, *n.a.* not applicable.

### Microscopic analysis of transepithelial migration

**Figure 2:**
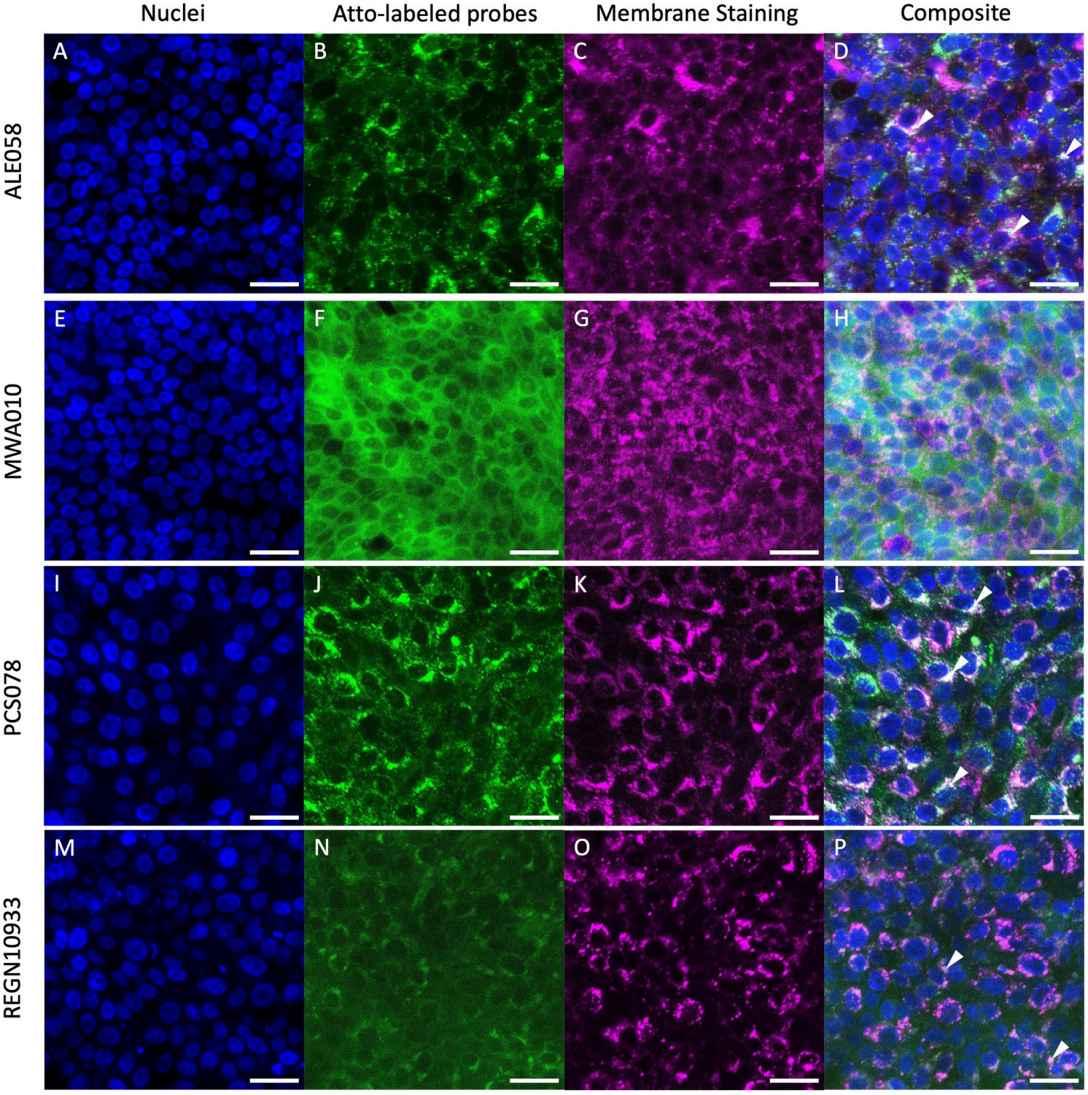
Confocal Laser Scanning Microscopy (CLSM) of reconstituted HBE incubated with fluorescence-labeled DARPins or control antibody. Multivalent DARPin ALE058 (**A-D**), monovalent HSA-binding DARPin domain MWA010 (**E–H**), negative control DARPin PSC078 (**I–L**) and REGN10933 antibody as positive control (**M–P**) were added to the basal cell culture medium together with the nuclear dye Hoechst 33342 and lipophilic membrane dye CellBrite^®^ Red. Cell cultures were fixed at 24 h with 4% formaldehyde for later imaging. Columns from left to right show nuclei in blue, Atto-488 labeled candidate and control molecules in green, membranes in magenta, and the overlays. The superposition of the green fluorescent signal of the Atto-488 labeled molecules with the red signal of the membrane dye results in white in the composite images (arrow heads). Scale bars: 25 μm.

The evaluation by Confocal Laser Scanning Microscopy (CLSM) confirmed the migration of the tested fluorophore labeled compounds through the intact bronchial epithelia. Live cell imaging (LCI) revealed intracellular fluorescence as early as 2 h of incubation (Fig. S1 in the Supplementary Information). Imaging of HBE incubated with the penta-valent DARPin candidate ALE058, the not SARS-CoV-2-binding controls PSC078 and MWA010, or with the monoclonal antibody REGN10933 at 160 µg/mL and for 24 h, confirmed efficient migration of all tested molecules across the epithelia. Figure 2 shows that at 24 h, all DARPins and the antibody can be found within the intact HBE. The smallest DARPin, MWA010, shows a strong, diffuse, green fluorescence signal that is particularly intense in areas likely corresponding to the intercellular space (Fig. 2F). The green fluorescence signals of the larger DARPin construct ALE058 (Fig. 2B) and its isotype control PSC078 (Fig. 2J) show a pattern, which is largely congruent with the magenta of the membrane staining (Fig. 2C,K), without any indication of considerable intercellular localization and with clear nuclear sparing. The fluorescence signal of the monoclonal anti-SARS-CoV-2 antibody REGN10933 (Fig. 2N) is comparable to that of the larger DARPins, however, with weaker intensity.

### Protection from SARS-CoV-2 infection

**Figure 3:**
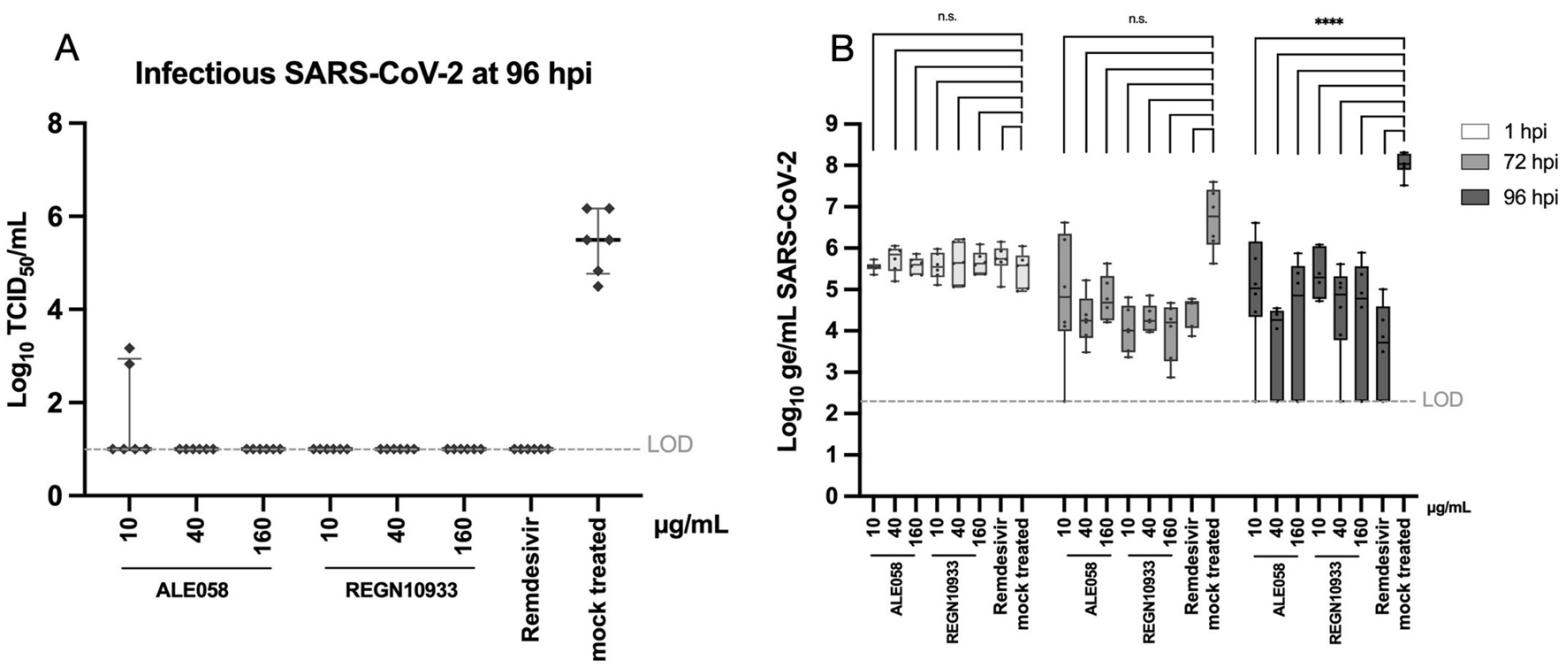
Antiviral activity of ALE058, REGN10933 and Remdesivir in HBE infected with SARS-CoV-2. HBE cultures were treated with either (i) ALE058 or REGN10933 at 10, 40 or 160 µg/mL, (ii) Remdesivir at 5 µM, or (iii) mock treated from the basolateral side, 2 days before infection with 10^4^ TCID_50_ SARS-CoV-2 from the apical side. The protective capacity of the compounds was assessed by (**A**) determining the infectious virus titer (TCID_50_/mL) in apical washes with a limiting dilution assay at 96 hpi, with the limit of detection (LOD) at 10 infectious particles/mL, and (**B**) measuring the viral genome equivalents/mL in apical washes by qRT-PCR at 1, 72 and 96 hpi. Results are shown as boxplots of 6 individual cell cultures (all orginating from the same donor lung) from 2 separate experiments. Boxes display interquartile ranges with the central bar indicating the median and whiskers minimal and maximal values. ****p < 0.0001; *n.s.* not statistically significant, *LOD* limit of detection.

The pre-incubation of HBE with the DARPin molecule ALE058 or the monoclonal antibody REGN10933 in the basal medium conferred good protection from epithelial infection with SARS-CoV-2 added to the apical, air-exposed side. While SARS-CoV-2 replicated in mock treated HBE to high Tissue Culture Infectious Dose 50 (TCID_50_) titers of 10^6^ TCID_50_/mL at 96 h post infection (hpi), there was no infectious virus retrieved from the apical surface of HBE treated with ALE058 at 40 µg/mL and 160 µg/mL, REGN10933 at 10 µg/mL, 40 µg/mL and 160 µg/mL, or Remdesivir at 5 µM (Fig. 3A). In four out of the six HBE cultures treated with 10 µg/mL ALE058, there was no virus detected in their apical washes either, while the remaining two apical washes contained low viral titers (10^2^–10^3^ TCID_50_/mL).

The monitoring of viral genome copies by quantitative reverse transcription polymerase chain reaction (qRT-PCR; Fig. 3B) showed the mean viral genome equivalents (ge) in apical washes of mock treated HBE to increase from 4.4 × 10^5^ ge/mL shortly after the infection (1 hpi) to 1.3 × 10^7^ ge/mL at 72 hpi. In contrast, in HBE pretreated with ALE058, REGN10933 or Remdesivir, there was a decrease of mean viral genome equivalents of 0.5–1.3 log between 1 and 72 hpi, except for HBE treated with 10 µg/mL ALE058, where the mean viral genome equivalents slightly increased. We did not observe any relevant correlation between the concentration of ALE058 or REGN10933 and the antiviral activity measured by qRT-PCR. The mean viral genome copy numbers were in the same order of magnitude for all treatments apart for the above mentioned treatment with the lowest concentration of ALE058, where there was a considerable variation of the data points (see Fig. 3B).

While the number of viral genome equivalents in apical washes of mock treated HBE further increased by 1 log over the following 24 h, it remained at the same level in HBE treated with 40 µg/mL and 160 µg/mL ALE058 or with 5 µM Remdesivir. There was a low to moderate increase in the number of viral genome equivalents for HBE pre-treated with REGN10933 at all tested concentrations from 72 to 96 hpi (Fig. 3B). At 96 hpi, the mean viral genome equivalents were in the same order of magnitude for all treatment groups and significantly different to the mock treated HBE (*p* < 0.05). In one or two out of six HBE cultures of each treatment group, the genome copy number was below the detection limit of 200 ge/mL.

The migration of macromolecules, such as DARPin therapeutics, through pulmonary epithelia is so far poorly understood. The present study in ALI cultures of reconstituted HBE demonstrates that antibodies and different DARPin candidates are able to traverse human bronchial epithelia at a comparable rate. In general, the amount of macromolecules reaching the apical side of HBE was found to depend on the amount applied to the basal side of the epithelium. For the DARPin candidates tested, transepithelial migration appears to be size dependent, as smaller DARPins were found to pass the epithelium more efficiently than the larger molecules. The mAb with twice the molecular weight of the therapeutic DARPin ALE058 (145 kDa versus 78 kDa) translocated efficiently to the apical side of the epithelium, at an approximately one log higher rate than ALE058. These different transepithelial migration rates did not translate into significant differences in the protective capacity of the two compounds at the tested concentrations (Fig. 3). Significant protection from SARS-CoV-2 infection was conferred by all three antiviral compounds at three different concentrations, as compared to mock treated HBE at 96 hpi. This confirms that therapeutically relevant doses of the antiviral DARPin ALE058, the mAb REGN10933 and the nucleoside analogue Remdesivir successfully penetrated the intact epithelial layer to protect the cells on the apical side, repressing viral replication. Unlike the other tested therapeutic compounds, Remdesivir is not a protein. Therefore, its transepithelial transport is likely different, but was not further investigated in this study.

Quantification of the compound concentration in the apical airway surface liquid of HBE is experimentally limited due to its unknown thickness and volume and, hence, of the dilution factor in the phosphate buffered saline (PBS) wash solution. As all HBE originated from the same donor and were processed in parallel, all airway surface liquids were assumed to be constant for comparison of the different tested settings and compounds. The data demonstrate size- and concentration-dependent migration of the different DARPin constructs across intact HBE, with the highest concentration in the apical wash of the smallest DARPin MWA010. This is in line with data from previous studies showing monomeric DARPins to rapidly clear from the blood and to penetrate into tumor masses instead of only accumulating on the surface of the tumor, as it is often observed for other macromolecules^10,16^. Thus, when comparing transepithelial migration rates of the monomeric DARPin MWA010 with the multimeric DARPins ALE058 and PSC078, each consisting of five DARPin elements, the observed difference in transepithelial migration rate is not unexpected. The high apical concentration of the monomeric DARPin is likely explained by at least partial paracellular migration, as supported by the fluorescence pattern revealed by CLSM analysis. The diffuse distribution pattern indicates that the smallest DARPin did not depend on vesicle transport to pass the epithelial barrier. In contrast, the granular fluorescence pattern found for the two larger DARPin constructs and the mAb REGN10933, as well as their co-localization with the red signal from the lipid staining indicate that these compounds are transported across HBE in a vesicle dependent manner. While transport mechanisms of antibodies have been extensively studied e.g. Refs.^13,17,18^, there is hardly any knowledge on the transport of DARPins across an intact airway epithelium. To our knowledge, it is not known to which extent DARPins may enter cells and what mechanisms are involved in transport into cells or across epithelia. Generally, proteins are thought to pass pulmonary epithelia by endocytic processes including receptor-mediated endocytosis (clathrin-coated pits or caveolae) and pinocytosis, i.e. by nonspecific, fluid-phase endocytosis, while for small proteins below a size of 40 kDa paracellular transport may dominate^19,20^. This corresponds with the fluorescence pattern seen in CLSM, indicating paracellular transport of the smallest candidate, MWA010, with a size of 14 kDa, while the two larger DARPin constructs in the size range of 80 kDa appear to pass the intact epithelium predominantly by transcytosis. Our data of course only apply in the context of intact airway epithelia. Inflammation and epithelial damage as seen during SARS-CoV-2 infection can cause disruption of the epithelial barrier and may allow for significant paracellular transport of larger proteins such as for the penta-valent DARPin construct ALE058 or monoclonal antibodies.

Both IgG and HSA have been shown to bind to FcRn, which protects these molecules from cellular catabolism and guides them into transcytosis pathways^12,17^. The FcRn is not only found in syncytiotrophoblast cells of the placenta, but also in various other cell types, such as enterocytes, endothelial cells, alveolar macrophages and bronchial epithelial cells^18,21^. Hence, FcRn binding allows IgG and HSA to pass epithelial barriers by vesicular transcytosis^17^. The FcRn binding is likely mediating efficient transport of the mAb through HBE as observed in the present study. Since FcRn has been shown to be involved in HSA transcytosis, we hypothesized it might also be involved in the transepithelial transport of HSA-bound DARPins. Interestingly, the ELISA results show a lower transepithelial transport for both DARPin constructs containing HSA-binding domains, when HSA was added to the basal cell culture medium (Fig. 1F). The resulting increase in size of the DARPin/HSA complex, or the addition of HSA to HBE, appear to reduce transepithelial transport efficacy, not supporting the hypothesis of enhanced transepithelial transport via transcytosis by HSA-binding. This suggests a tradeoff between a prolonged systemic half-life mediated by a HSA-binding domain, i.e. the serum albumin recycling via the FcRn receptor^11,12^, and the reduced transepithelial transport rate observed in HBE due to the increased size of the DARPin/HSA complex.

## Conclusions

In summary, the findings on the ability and efficacy of DARPin candidates to pass HBE and potentially other cell membrane layers in lungs or other organs are comparable to those of the well-characterized therapeutic mAb REGN10933. This matches with preclinical and clinical efficacy data for the antiviral SARS-CoV-2 DARPin candidate ensovibep, where improved outcomes for early-stage ambulatory patients in the range of anti-SARS-CoV-2 mAb candidates was observed. Taken together, this encourages potential further development of DARPin molecules for pulmonary diseases.

## Methods

### Summary of experimental procedure

In this study, we investigated the transepithelial migration of a DARPin construct specific against the SARS-CoV-2 spike protein (ALE058) and its antiviral efficacy. The transepithelial transport of ALE058 was compared to further DARPin formats and to the anti-SARS-CoV-2 mAb REGN10933, and assessed by ELISA and CLSM. In addition, efficacy of protection against authentic, original SARS-CoV-2 by ALE058 was compared to REGN10933 and Remdesivir. For this purpose, HBE were incubated with the therapeutic candidates before infection with SARS-CoV-2. Antiviral efficacy was assessed based on viral titers and genome equivalents.

### DARPin generation (ALE058, MWA010, PSC078)

The DARPin constructs, selected and cloned as described in Rothenberger et al.^4^, were transformed in *E.coli* BL21 cells, plated on Luria Bertani (LB) agar prepared from LB powder (Pronadisa, Madrid, Spain) and agar (Invitrogen, Schlieren, Switzerland), containing 1% glucose (Roth, Arlesheim, Switzerland) and 50 μg/mL ampicillin (Roth), and then incubated overnight at 37 °C. A single colony was picked into Terrific Broth (TB) medium (e.g. Ref.^22^, containing 1% glucose and 50 μg/mL ampicillin) and incubated overnight at 37 °C, shaking at 230 rpm. Fresh TB medium (containing 50 μg/mL ampicillin) was inoculated with 1:20 of the overnight culture and incubated at 37 °C and 230 rpm. At an optical density of 1.1 at a wavelength of 600 nm (OD_600_), the culture was induced by addition of isopropyl β-D-1-thiogalactopyranoside (IPTG; Biosolve Chimie, Dieuze, France, 0.5 mM final concentration) and incubated further for 5 h at 37 °C and 230 rpm. Bacteria were harvested by centrifugation (10 min, 5000 × *g*). After cell disruption by sonication, primary recovery was performed by heat treatment for 30 min at 62.5 °C and subsequent centrifugation (15 min, 12,000 × *g*). Thereafter, 20 mM Imidazole (Merck, Taufkirchen, Germany) and 1% Triton X-100 (LubioBioscience, Zurich, Switzerland) were added to the supernatant. The 0.22 µm-filtered supernatant was then purified by immobilized metal affinity chromatography (IMAC, HisTrap FF crude, Cytiva, Uppsala, Sweden) using a N-terminal His-tag and including a wash step with 1% Triton X-100 and a step elution with 250 mM Imidazole. Subsequently, the elution fraction from the IMAC step was applied on a size exclusion chromatography column (Superdex 200, Cytiva). Fractions of interest were then pooled and concentrated. The final sample was filtered through a 0.22 µm Mustang E filter (PALL Corporation, New York, USA) for endotoxin removal and sterile filtration before quality control.

### Generation of monoclonal anti-spike IgG1 antibody Casirivimab (REGN10933)

Publicly available sequences of variable domains of the monoclonal antibody REGN10933 were used to synthetize the corresponding cDNA fragments and cloned into a proprietary expression vector at Evitria AG (Schlieren, Switzerland). Generated vectors containing the constant immunoglobulin chains were used for transfection in Chinese hamster ovary cells by Evitria. Sterile filtered cell supernatants were purified via affinity purification with a HiTrap MabSelect column (Cytiva) followed by size exclusion chromatography using a HiLoad 26/600 Superdex 200 column (GE Healthcare, Danderyd, Sweden) in PBS, pH 7.4. Selected fractions were pooled and quality controlled by Sodium Dodecyl Sulphate–Polyacrylamide Gel Electrophoresis (SDS-PAGE), size exclusion chromatography and endotoxin measurement before use in assays.

### Cell cultures

Reconstituted HBE cultures were prepared according to approved protocols^23–25^. HBE cells were isolated from lungs provided by the Midwest Transplant Network (Kansas City, KS, USA). The University of Kansas Institutional Review Board determined that the consent of organ donation for research done by the organ procurement agency covers research use of this material. The lungs were obtained from a deceased individual with minor and deidentified information, and therefore their use does not constitute human subjects research as defined by Code of Federal Regulations 46.102. The here presented research was carried out in accordance with the relevant guidelines and regulations. As illustrated in Fig. 1A, HBE cells were grown and differentiated at a permanent ALI on microporous 6.5-mm Transwell^®^ inserts (i.e. 24-well inserts with a growth area of 0.33 cm^2^; Corning, Vitaris, Baar, Switzerland) coated with human placental collagen Type IV (Merck). Serum-free ALI medium was prepared in house following a previously published protocol^23,26^. Basal medium was changed, and mucus removed by washing cell surfaces with Dulbecco’s phosphate-buffered saline (DPBS, pH 7.4, Corning, Root, Switzerland) every other day. Differentiation was assessed by the presence of beating cilia and mucus secretion, typically visible after 21 days of culture at ALI.

### Quantification of the transepithelial migration of DARPin candidates and a control antibody by ELISA

DARPins with different numbers of binding domains and a control antibody, as described in Table 1, were added to the basal medium of HBE cultures at concentrations of 10, 40 and 160 µg/mL. Since the two DARPin compounds ALE058 and MWA010 contain HSA-binding domains, the cell culture medium was supplemented with 10 µM HSA (Albumin CSL 20%, CSL Behring, Bern, Switzerland), i.e. at physiological concentration, to saturate the HSA-binding domains of the DARPin candidates. Controls comprised of HBE incubated in cell culture medium containing HSA only, i.e. without DARPins or antibody. The impact of HSA bound to the DARPin was tested by using cell culture medium without HSA at the highest concentration of 160 µg/mL for the DARPins containing HSA-binding domains.

Transepithelial trafficking of DARPins and the control antibody was evaluated by ELISA at 24 h post inoculation by measuring the concentrations of the various compounds in the basal medium and in apical washes of the HBE cultures, as well as within cell lysates (Fig. 1A). Basal cell culture medium (500 μL) was collected and apical surfaces were washed with 200 μL PBS for 10 min. To generate cell lysates, 100 μL of a lysis buffer (cytotoxicity detection kitPLUS; Roche Diagnostics AG, Rotkreuz, Switzerland) was apically added for 30 min at room temperature (RT) before freezing the cell cultures at -80 °C alongside with the apical washes and basal media. Molecules in the different assay compartments were quantified using the following ELISA procedures.

For detection of the DARPin or mAb molecules, 50 µL of PBS containing either 10 nM polyclonal goat-anti-rabbit-IgG (ThermoScientific, Schlieren, Switzerland) for the DARPin set-up, or 66 nM Neutravidin for the mAb set-up was coated onto 96-well Nunc MaxiSorp ELISA plates (ThermoScientific) and incubated for 16 h at 4 °C. The wells were washed with 300 μL of PBS + 0.1% Tween20 (PBST, Merck) three times and blocked with 300 μL PBST + 0.25% Casein (PBS-T-C, Sigma) for 2 h at RT and shaking at 300 rpm. The plate was then washed three times with 300 μL PBST. As capture antibody for the DARPin molecules, 50 µL of 5 nM monoclonal rabbit-anti-DARPin antibody in PBS-T-C was added to the plate and incubated for 1 h at RT and 300 rpm. Wells were washed three times with 300 μL PBST. For capturing of the mAb, 50 µL of 10 nM biotinylated SARS-CoV-2 Spike RBD (ACRO Biosystems, Newark, USA) in PBS-T-C was added per well and incubated for 1 h at RT and 300 rpm. Then, the wells were washed three times with 300 µL PBST.

For analysis, the samples were thawed at RT and mixed by pipetting. In a dilution plate (Greiner Bio-One, Kremsmünster, Austria), threefold serial dilutions of the samples with ten steps starting with a 1:10 dilution in PBS-T-C were prepared. In parallel, a threefold serial dilution with ten steps of the standard starting with 20 nM in PBS-T-C was prepared. 50 µL of each sample dilution and standard were transferred to the ELISA plate and incubated for 1 h at RT and 300 rpm. Plates were washed three times with 300 μL PBST. Detection antibody conjugated with horseradish peroxidase (for DARPin detection: anti RGS-His-HRP, Qiagen, Germantown, USA; for mAb detection: anti human IgG-HRP, A0170, Merck) was diluted at 1:10 000 in blocking buffer, and 50 μL was added to each well and incubated for 1 h at RT and 300 rpm. Plates were washed three times with PBST, then incubated with 1-Step™ TMB-ELISA Substrate Solution (BUF062C, Biorad, Hercules, USA) for 5 min, prior to stopping the reaction with 1 M sulfuric acid. Plates were then read for absorbance at 450 and 650 nm (TECAN Sunrise, TECAN, Maennedorf, Switzerland) within 30 min of stopping the reaction. Absorbance (OD) is calculated as the absorbance at 450 nm minus the absorbance at 650 nm to remove background prior to data analysis.

### Confocal laser scanning microscopy

Fluorophore labeling of DARPin candidates and control molecules was achieved by labeling either the thiol groups via a C-terminally incorporated cysteine for MWA010 or the primary amine groups for ALE058, PSC078 and REGN10933 following the user manuals provided (Jena Bioscience, Germany). Residual free Atto-488 was removed by preparative size exclusion chromatography using a Superdex 200 150 GL (Cytiva) at a flowrate of 0.5 mL/min with an injection volume of 100 µL and the 0.1 min eluent fractions were collected. This process was performed under low light conditions to avoid fluorophore bleaching. Maintained binding to the SARS-CoV-2 spike protein and to HSA (where HSA-binders were incorporated, i.e. MWA010 and ALE058) was verified by ELISA for all labeled binders, in comparison to the respective non-labeled molecule.

The Atto-488 labelled compounds were added at a concentration of 160 μg/mL to the basal medium of HBE (supplemented with HSA) for 24 h. Simultaneously, nuclear staining solution (Hoechst 33342; Merck) at a dilution of 1:2000 and 5 μL CellBrite Red Cytoplasmic Membrane Dye (Chemie Brunschwig, Basel, Switzerland) per 500 μL were added to the basal medium. LCI of the cultures was performed at 2, 7 and 24 h. After 24 h, the HBE cultures were chemically fixed in 4% formaldehyde.

For imaging HBE on their membrane support, the cell culture inserts were placed on glass bottom petri dishes (MatTek Europe, Bratislave, Slowakia). CLSM was performed with a ZEISS LSM 880 with Airyscan (Carl Zeiss Microscopy GmbH, Munich, Germany) using a 63 × oil immersion objective. Image processing and 3D reconstruction were performed using the Fiji ImageJ software^27^.

### Protection assay

The DARPin (ALE058) and the mAb (REGN10933) were diluted in basal medium supplemented with 10 μM HSA (CSL Behring) to concentrations of 10, 40 and 160 µg/mL. Remdesivir (Gilead Sciences, Inc., Foster City, CA, USA) was used as positive control, at a concentration of 5 µM. At 48 h prior to infection, all inserts were rinsed twice with DPBS and basal medium was exchanged to the diluted molecules in triplicates. For the infection, 1 × 10^4^ tissue culture infectious dose 50 (TCID_50_) of SARS-CoV-2 (2019-nCoV/IDF0372/2020) diluted in DPBS at a volume of 100 µL was added to the apical compartment, and cell cultures were incubated at 37 °C, 5% CO_2_ and > 85% relative humidity (rH) for 1 h. Subsequently, the virus inoculum was removed, the inserts were washed once with DPBS, and the basal medium was renewed with fresh medium containing the tested therapeutics. To quantify apical virus release, the apical side of the HBE was washed at 72 and 96 hpi with 200 µL DPBS for 10 min at 37 °C. To analyze extracellular viral RNA, 100 µL of the wash was inactivated, extracted and eluted in 100 μL elution buffer using the MagNAPure 96 system (Roche, Basel, Switzerland). Viral RNA was quantified by qRT-PCR using 5 μL eluate and the TaqMan™ Fast-Virus-1 Step master mix (Life Technologies, Zug, Switzerland), targeting the SARS-CoV-2 non-structural protein 14 (nsp14)^28^ on the LightCycler 96 system (Roche). Genome equivalents (ge) per milliliter were calculated using an in-vitro synthesized RNA standard. Infectious virus titer at 96 hpi was determined by incubation of serial ten-fold dilutions of apical washes in Dulbecco's Modified Eagle Medium (DMEM) containing 2% fetal bovine serum (Bioswisstec, Schaffhausen, Switzerland) for three days at 37 °C, > 85% rH and 5% CO_2_ on Vero E6 cells expressing TMPRSS2^29,30^ (obtained from the Centre for AIDS Reagents, National Institute for Biological Standards and Control, Potters Bar, United Kingdom). After crystal violet staining, virus titers were calculated with the Spearman-Karber method^31,32^ and reported as TCID_50_/mL.

### Statistics

Calculated genome numbers obtained by qRT-PCR were analyzed using a two-way analysis of variance (ANOVA) with Dunnett's multiple comparisons test against mock treatment. A *p*-value ≤ 0.05 was considered statistically significant.

## Supplementary Information

Supplementary Figure S1.

## Data Availability

All data generated or analyzed in this study are included in this published article (and its Supplementary Information files).
