# Supplementary material for: Transport of Designed Ankyrin Repeat Proteins through reconstituted human bronchial epithelia and protection against SARS-CoV-2
Source: Sci Rep. 2023 Apr 4;13:5537. doi: 10.1038/s41598-023-32269-1 (PMC10072008; doi:10.1038/s41598-023-32269-1)
Supplement: Supplementary file 1 — Supplementary Figure S1. [file 41598_2023_32269_MOESM1_ESM.docx]

**Supplementary Figure**

**Figure S1. Live cell imaging (LCI) of transepithelial transport of ALE058 at 2, 7 and 24 h.** HBE were incubated with Atto-labeled ALE058 in the basal cell culture medium for 24 h, Hoechst 33342 was added for nuclear staining. LCI was performed at 2 h (**A-C**), 7 h (**D-F)** and 24 h (**G-I**). Columns from left to right show nuclei in blue, Atto-488 labeled ALE058 in green, and overlays. Scale bars: 25 μm.
